# Nasopharyngeal carriage of *Streptococcus pneumoniae* and antimicrobial susceptibility pattern among school children in South Ethiopia: post-vaccination era

**DOI:** 10.1186/s13104-019-4330-0

**Published:** 2019-05-29

**Authors:** Fiseha Wadilo Wada, Efrata Girma Tufa, Tezera Moshago Berheto, Fithamlak Bisetegen Solomon

**Affiliations:** 1 School of Life Science, Addis Ababa University, Addis Ababa, Ethiopia; 2 School of Public Health, Departement of Reproductive Health and Human Nutrition, Wolaita Sodo University, Wolaita Sodo, Ethiopia; 3 Medical Laboratory Department, College of Health Science and Medicine, Wolaita Sodo University, Pobox 138, Sodo, Ethiopia; 4 School of Public Health, College of Health Science and Medicine, Wolaita Sodo University, Sodo, Ethiopia

**Keywords:** Nasopharyngeal, Carriage, *S. pneumoniae*, Sodo Zuria Woreda, Antibiotic, School children

## Abstract

**Objective:**

The aim of this study was to investigate nasopharyngeal carriage rate and antibiotic susceptibility patterns of *Streptococcus pneumoniae* among school children.

**Results:**

Three hundred eleven (43.8%) became culture positive for *S. pneumoniae.* The carriage rate among children, 3–5 years old was 62.5%, which was higher than the carriage rate of 38.6% among 6–13 years old children. Age ≤ 5 years and co-sleeping with siblings remained significantly associated with *S. pneumoniae* carriage. 155 (49.8%) of the isolates were resistant to co-trimoxazole, 152 (48.9%) of the isolates were resistant to tetracycline, and 88 (28.3%) of isolates were resistant to oxacillin. Multi drug resistant *S. pneumoniae* was observed in 90 (28.9%) of isolates. There is high prevalence of *S. pneumoniae* in primary school children in our study area. Relatively high carriage rate of resistance to oxacillin, tetracycline and co-trimoxazole were observed. These findings provide baseline data for future studies to further compare pneumococcal carriage rates and antibiotic resistance patterns.

**Electronic supplementary material:**

The online version of this article (10.1186/s13104-019-4330-0) contains supplementary material, which is available to authorized users.

## Introduction

World health organization (WHO) estimates that ~ 1.6 million people, including up to 1 million children aged < 5 years, die of invasive pneumococcal disease every year, with developing countries bearing the greatest burden [1–4]. *Streptococcus pneumoniae* is a leading cause of childhood disease worldwide [5] which is responsible for 30% of pneumonia associated deaths in such settings [6].

*Streptococcus pneumoniae* is the leading cause of pneumonia in children and the most common cause of morbidity among infectious agents in Ethiopia [7]. Each year, 104,000 under five deaths are due to pneumococcal infections who are not vaccinated [8, 9]. The prevalence of nasopharyngeal carriage of *S. pneumoniae* varies according to age, geographic area, crowding, concomitant respiratory tract illness and antibiotic consumption. Prospective studies have revealed considerable intra-individual changes in nasopharyngeal carriage rates of *S. pneumoniae* over time [10–12]. The percentage of carriers also increases significantly in schools, orphanages, military camps and persons with chronic respiratory diseases [13].

Multidrug-resistant (MDR) *S. pneumoniae* clones have already spread throughout the world due to nasopharyngeal colonization over the last few years [10]. Antibiotic-resistant *S. pneumoniae* infections may require higher doses of antibiotic, longer duration of treatment and hospitalization, the use of more expensive medications, or use of medications with greater side-effect potential [14].

Because of the highest frequency of pneumococcal colonization and the highest crowding index are found in young children, this group is the most important vector for horizontal dissemination of pneumococcal strains within the community. Part of the strategy to prevent pneumococcal disease focuses on prevention of nasopharyngeal colonization [10], accurate diagnosis and effective antimicrobial chemotherapy [15].

Early nasopharyngeal acquisition of *S. pneumoniae* is associated with both high carriage in young infants and a high rate of pneumococcal disease [16]. Since nasopharyngeal carriage is the necessary first step in the pathogenesis of pneumococcal disease [17], knowledge about the carriage rate is the necessary first step for effective prevention and control of pneumococcal disease.

Even after the introduction of Pneumococcal conjugate vaccine (PCV-10) in Ethiopia, *S. pneumoniae* mortality among under fives is still increasing [18]. PCV effectively induces protection against nasopharyngeal carriage of vaccine type pneumococci in young children as well as in older age groups, it is necessary to know the current carriage status of *S. pneumoniae* in order to allow evaluation on the role of vaccination, to determine risk factors for *S. pneumoniae* carriage and to assess the burden of antibiotic resistance on these strains.

## Main text

### Methods

Institution based cross-sectional study was conducted in selected schools of Sodo Zuria Woreda of Wolaita Zone from Sept 5 to Dec 25, 2016. Sodo Zuria Woreda is one of the 12 districts in Wolaita zone. It has 31 kebeles (the smallest administrative structure in Ethiopia) and 7 governmental health centers. The total population is about 200,866. Sodo Zuria District consists of 43 primary schools. Within the town and the district, six primary schools namely, Shola Kuto, Waja Kero, Ofa, Gandeba, Delbo wogene and Waraza Gerera from Sodo zuria district were selected as the study schools.

All children in primary school who are ≤ 13 years of age were considered as the source population; and the study population was randomly selected primary school students below grade five. Students with wounded nose (nasal trauma and injuries) and previous history of infection who take antibiotics were excluded from the study.

The sample size was determined based on single population proportion formula by assuming a nasopharyngeal carriage of 88% according to a study in Gambia [19]. The expected margin of error (d) was 0.05 and the confidence interval (Zα/2) was 95%. With the above sampling parameter and a 10% contingency for possible non-respondents we came up with a sample size of 357. To improve the power of the test the sample size was further doubled and the final sample size was 714 primary school children.

A two-stage sampling technique was employed to draw a representative sample of 714 school aged and preschool children. Then lists of all primary schools having any of under 5th grades were identified by the help of the district education officials. Overall 25 schools, all children under the 5th grade were identified. A total of six schools were selected using probability proportional to size of enrolment. Children were allocated based on probability allocation to the size of each selected school, and participants were selected from the sampling frame.

The selection of primary schools was based on probability proportion to size (PPS); the number of schools required from the district six to derive the sampling interval (SI) divided the total population of attendants in each school. The first cluster was selected by multiplying the sampling interval by a random computer generated three-digit decimal number between 0 and 1. The resulting number was traced in the cumulative population column, and the first cluster was taken from the corresponding school. The following clusters were identified by adding the sampling interval to the previous number.

Questionnaires were completed by interviewing the parents (guardians). Clinical data, the medical history of the children and associated risk factors were collected by trained nurses in collaboration with kindergartens.

Nasopharyngeal specimens were collected by laboratory technicians using rotating cotton-tipped flexible swabs; during the smooth insertion through the nostrils of each individual, whose head was tipped backward; the swabs were left in the posterior nasopharynx for 5 s to saturate the tip. Once a swab specimen was collected, it was placed in a tube of skim milk–tryptone–glucose–glycerin (STGG) transport medium and transported to the WSU microbiology laboratory within 1 h of collection for processing.

A loopful nasopharyngeal specimen from STGG tube was inoculated onto colombia agar (Oxoid, England) on plates containing 5% sheep blood and 5 µg/ml gentamicin. The inoculated plates were incubated for 24 h at 35–37 °C in an atmosphere of 5–10% CO_2_ using a candle jar. *S. pneumoniae* was identified by its colony morphology, haemolytic activity, optochin susceptibility and bile solubility [20]. Zones of inhibition > 14 mm indicate susceptibility, 7 to 13 mm are indeterminate and a zone < 7 mm is resistant to optochin when a 6 mm size disc is used and the culture is incubated in 5% CO_2_ [21].

The Kirby–Bauer disc-diffusion test was used to determine antibiotic susceptibility testing. A sterile cotton swab was dipped into the standardized solution of bacterial cultures and used for evenly inoculating onto Muller–Hinton agar (Oxoid, England) plates containing 5% sheep blood and allowed to dry. The plates were incubated at 37 °C for 24 h, and the diameters were measured [22]. Isolates were classified as susceptible, intermediately resistant or resistant to the antibiotics based on the recommendations of Clinical Laboratory Standard Institute (CLSI) 2014.

*Multidrug resistant* defined as acquired non-susceptibility to at least one agent in three or more antimicrobial categories [23].

The quality of transport media, culture media and antimicrobial susceptibility was checked using a standardized reference strain of *S. pneumoniae* ATCC 49619.

The data was edited, cleaned, and restructured in Epidata analysis v2.2 then exported to SPSS version 21, for analysis. Bivariate analysis and multiple logistic regressions were used. P value less than 0.05 was used to declare statistical significant association.

### Result

#### Socio-demographics information

710 out of 714 children were involved in the study. The age of these children ranges from 3 to 13 years giving the mean age of 8.06 (95% CI (7.88, 8.244), 50.3% of the children were females. (Additional file 1: Table S1).

#### Nasopharyngeal carriage rate

311/710 (43.8%) children were positive for *S. pneumoniae.* The carriage rate among children 3–5 years old was 62.5%, which was higher than the 38.6% carriage rate among 6–13 year old children (P = 0.02). The carriage rate in male and female children were 42.1% and 43.1% respectively, which did not show significance difference between genders (P = 0.85) (Table 1).

**Table 1:** Nasopharyngeal carriage rate of *S. pneumoniae* in relation to age and sex, Sodo Zuria Woreda, South Ethiopia, 2014

Age	Carriage status (n = 311)				
	Male		Female		Total (%)
	Positive (%)	Negative (%)	Positive (%)	Negative (%)	
3–5 years	28 (9)	18 (9)	45 (28.3)	29 (14.6)	120 (16.9)
6–13	124 (39.9)	183 (91)	114 (71.7)	169 (85.4)	590 (83.1)
Total	152 (21.4)	201 (28.3)	159 (22.4)	198 (27.9)	710 (100)

#### Risk factors associated with S*. Pneumonia* carriage

Age ≤ 5 years and slept with siblings remained significantly associated with *S. pneumoniae* carriage. The odds of carrying pneumococci were approximately twice as high among those children (Table 2).

**Table 2:** Risk factors associated with nasopharyngeal carriage of *S. pneumonia* among school children, Sodo Zuria Woreda, South Ethiopia, 2014

Associated factors	Carriage rate					
	No (399)	Yes (311)	P-value	COR (95% CI)	P-value	AOR (95% CI)
Male	201	152	0.85	0.96 (0.62–1.48)		
Age ≤ 5 years	47	73	0.00	2.64 (1.46–4.78)	0.02	2.09 (1.13–3.87)
Having at list one sibling	356	284	0.50	1.22 (0.50–2.53)		
Living with one or more siblings < 6-years old	217	182	0.04	1.61 (1.03–2.51)		
Co-sleeping with siblings	229	233	0.00	2.25 (1.41–3.60)	0.01	1.89 (1.15–3.09)
Passive smoking	42	63	0.01	2.39 (1.30–4.39)		
Income < 500 birr/month	127	114	0.21	1.33 (0.85–2.08)		
Have one room in the house	89	78	0.30	1.30 (0.79–2.15)		

*COR* crude odds ratio, *AOR* adjusted odds ratio

#### Antimicrobial susceptibility pattern

Resistance rate of 48.9% to tetracycline, 45.3% to co-trimoxazole and 28.3% to oxacillin were isolated. The lowest resistance rate was exhibited by chloramphenicol which was 12.5% (Additional file 2: Figure S1).

#### Antibiogram pattern

Out of 311 strains, 90 (28.9%) were multidrug resistant, 23 (10.9%) strains were resistant to 3 antimicrobials, nine isolates were resistant to 4 antimicrobials and 4 (2.1%) of the isolates were pan resistant (Table 3).

**Table 3:** Antibiogrampattern of *S. pneumoniae* (n = 311) among school children in Sodo Zuria Woreda, South Ethiopia, 2014

Frequency of resistance	Antibiotics pattern	No	%
R_0_	Susceptible to all drugs	97	31.2
R_1_	Oxacillin	12	3.9
	Tetracycline	24	7.7
	Co-trimoxazole	27	8.7
	Oxacillin/tetracycline	12	3.9
R_2_	Oxacillin/co-trimoxazole	17	5.4
	Tetracycline/co-trimoxazole	16	5.1
	Tetracycline/chloramphenicol	9	2.9
	Tetracycline/erythromycin	7	2.3
	Oxacillin/tetracycline/co-trimoxazole	36	11.6
R_3_	Tetracycline/co-trimoxazole/erythromycin	14	4.5
	Tetracycline/co-trimoxazole/chloramphenicol	8	2.6
	Co-trimoxazole/chloramphenicol/erythromycin	9	2.9
	Oxacillin/tetracycline/co-trimoxazole/erythromycin	10	3.2
R_4_	Oxacillin/tetracycline/chloramphenicol/erythromycin	9	2.9
R_5_	Oxacillin/tetracycline/co-trimoxazole/erythromycin/chloramphenicol	4	1.3

### Discussion

*Streptococcus pneumoniae* carriage rate in healthy children under 5 years of age ranged from 20 to 93.4% in low income countries which was generally higher than reported in lower-middle income countries (range 6.5–69.8%) [24]. The carriage rate of *S. pneumoniae* among children ≤ 5 years, 62.5%, in this study was corroborated with the cumulative prevalence in African countries 64.8% [24] and Kenya 56.7% [25] but higher than the reported carriage rates elsewhere [24–31]. A 38.6% carriage rate in children aged 5–15 years in the current study is in harmony with 41% reported in Kenya [32] but much higher findings, 53.6% for 5–10 years old children in northern India [33] and, > 80% for children aged 5–14 years in Gambia were also documented [3]. On the contrary, much lower prevalence, 8.2% for 10–19 years old children in Brazil [34] as compared with this study were noticed. Possible reasons for the difference in the carriage rate might be due to variation in specimen, age, geographic area, socio-demographic factors, the availability of PCV vaccine, and the study time.

In the literature, colonization rates were independently related to risk factors such as young age [30, 34–37], day care center attendance [27, 35] having young siblings [30, 35, 36], the number of bedroom occupants < 5 years of age [36] and exposure to passive smoke in the household [38]. In our study, nasopharyngeal carriage of *S. pneumoniae* was high on children ≤ 5 years old and on those who co-sleeping with his/her siblings. The decline of carriage rate as age increases could be as a result of decreased close contact among adults as compared with young children. However, it could also reflect acquisition of local mucosal immunity as a result of repeated colonization by many different serotypes [32].

Penicillin resistant in various studies has been reported to be as low as 2.2% [27] to as high as 83.5% [39]. Penicillin resistance rate in Ghana was 37.3% [40], which is nearly comparable with the current 28.3% result. High resistance to co-trimoxazole, 45.3% was noted in this study which was also evidenced across literature from different parts of the world [2, 33, 38, 41, 42]. In the contrary to this, much lower finding, 12.7% were documented from Zambia [15]. Seventeen percent resistance to erythromycin in the current study differed with no resistances elsewhere [2, 3, 40] to 96%, 77% and 33.5% resistance in Taiwan [38], Hong Kong [41], and Greece [4] respectively. In this study, we found 48.9% resistance to tetracycline, which is nearly comparable with 42.9% resistance from Spain [13]. A higher prevalence of tetracycline resistant *S. pneumoniae* isolates, 63.4% and 60.8% were reported from Ghana [40] and India [33] respectively. But in some other countries, lower tetracycline resistance rate of 32.3% [3], 26.4% [4], 23% [15] and 1% [43], were also reported. Low resistance prevalence to chloramphenicol were reported from African countries: 13.3% from Ghana [39], 6.3% from Gambia [3], 3.9% from Zambia [15] and 4% from Kenya [2], which is comparable with our 12.5% finding. But in contrast to our study, 33.7% was also reported from Hong Kong [41]. A wide variation in antibiotic resistance rate of *S. pneumonia* emphasizes the need for periodic local surveillance, in order to prevent and manage pneumococcal disease.

### Conclusion

Nasopharyngeal carriage of *S. pneumonia* among school children can be a potentially risk for children. Children ≤ 5 years age and co-sleeping with siblings play a role in pneumococcal carriage. Higher prevalence of resistance to oxacillin, tetracycline and co-trimoxazole was observed. Hence, nationwide surveillance for resistance pattern and serotype distribution in Ethiopia is necessary to guide the rational choice of antimicrobial agents.

## Limitation

Serotyping assessment was not conducted.

## Additional files

**Additional file 1: Table S1.** Socio-demographic characteristics of school children in Sodo Zuria Woreda, South Ethiopia, 2014.

**Additional file 2: Figure S1.** Antibiotic susceptibility pattern of *S. pneumoniae* strains, Sodo Zuria Woreda, South Ethiopia, 2014.

## Data Availability

The data that support the findings of this study are available. Anyone interested can get upon reasonable online request by writing to fitha2007@yahoo.com.

## Abbreviations

AOR: adjusted odds ratio
ATCC: American Type Culture Collection
CLSI: Clinical Laboratory Science Institute
COR: crude odds ratio
IPD: invasive pneumococcal disease
MDR: multi drug resistant
PCV: pneumococcal conjugate vaccine
RTI: respiratory tract infection
SPSS: Statistical Package For Social Science
STGG: skim milk–tryptone–glucose–glycerin
WHO: World Health Organization

## References

[1] World Health Organization (2007). Pneumococcal conjugate vaccine for childhood immunization—WHO position paper. WklyEpidemiol Rec..

[2] Nyandiko W, Greenberg D, Shany E, Yieannoutsos T, Musick B, Mwangi A (2007). Nasopharyngeal Streptococcus pneumoniae among under five year old children at the MOI teaching and referral hospital, eldoret, Kenya. E Afr Med J..

[3] Philip C, Abiodun A, Kawsu S (2006). Nasopharyngeal carriage of *Streptococcus pneumoniae* in Gambian Villagers. Clin Infect Dis.

[4] Ioannis K, Garyphallia P, Antonios A (2007). Nationwide surveillance of *Streptococcus pneumoniae* in Greece: patterns of resistance and serotype epidemiology. Int J Antimicrob Agents.

[5] Greenwood B (1999). The epidemiology of pneumococcal infection in children in the developing world. Philos Trans R Soc Lond B.

[6] Leowski J (1986). Mortality from acute respiratory infections in children under 5 years of age: global estimates. World Health Stat Q.

[7] Paola M, Susanna E, Gian S (2002). Nasopharyngeal carriage of *Streptococcus pneumoniae* in healthy children: implications for the use of heptavalent pneumococcal conjugate vaccine. EmergInft Dis..

[8] Clinton Foundation. Delivering lifesaving vaccines in Ethiopia. Available online: http://www.clintonfoundation.org/main/clinton-foundation-blog.html/2012/07/11/delivering-lifesaving-vaccines-in-ethiopia. Accessed 8 June 2016.

[9] O’Brien KL, Wolfson LJ, Watt JP, Henkle E, Deloria-Knoll M, McCall N, Lee E, Mulholland K, Levine OS, Cherian T (2009). Burden of disease caused by *Streptococcus pneumoniae* in children younger than 5 years: Global estimates. Lancet.

[10] Bogaert D, Groot R, Hermans P (2004). *Streptococcus pneumoniae* colonisation: the key to pneumococcal disease. Lancet Infect Dis.

[11] Paola M, Stefania G, Susanna E (2001). Seasonal variations in nasopharyngeal carriage of respiratory pathogens in healthy Italian children attending day-care centres or schools. J Med Microbiol.

[12] Ron D. Nasopharyngeal carriage of *Streptococcus pneumoniae*: epidemiology and influence of vaccination. Available http://findarticles.com/p/articles/mi_hb4384/is_10_43/ai_n42105977/?tag=content;col1. Accessed 17 May 2015.

[13] López B, Cima F, Vázquez A (1999). Epidemiological study of *Streptococcus pneumoniae* carriers in healthy primary-school children. Eur J ClinMicrobiol Infect Dis..

[14] Food, medicine and healthcare administration and control authority of Ethiopia. Standard treatment guidelines for General Hospital. 3rd edn, 2014; p. 313–4. http://www.fmhaca.gov.et/wp-content/uploads/2019/03/STG-General-Hospital. Accessed 5 Sept 2017.

[15] Woolfson A, Huebner R, Wasas A, Chola S, Godfrey-Faussett P, Klugman K (1997). Nasopharyngeal carriage of community-acquired, antibiotic-resistant *Streptococcus pneumoniae* in a Zambian paediatric population. Bull World Health Organ.

[16] WHO. GAVI Alliance partners to tackle childhood killer in Ethiopia. Available at: http://www.who.int/immunization/newsroom/press/introduction_pneumococcal_vaccine_in_Ethiopia/en/index.html. Accessed 5 Sept 2017.

[17] Christian L, Jeevan B, Subarna K (2009). Nasopharyngeal carriage of *S. pneumoniae* among young children in rural Nepal. Trop Med Int Health..

[18] Negera A, Abelti G, Bogale T, Gebreselassie T, Pearson R. An analysis of the trends, differentials and key proximate determinants of infant and under-five mortality in Ethiopia. Further analysis of the 2000, 2005, and 2011 demographic and health surveys; DHS further analysis Reports No 79; ICF International: Calverton, MD, USA, 2013. **(Google Scholar)**.

[19] Hill PC, Akisanya A, Sankareh K (2006). Nasopharyngeal Carriage of *Streptococcus pneumoniae* in Gambian Villagers. Clin Infect Dis.

[20] Monica C (2006). District laboratory practice in tropical countries.

[21] Osman A, Joyce N, Pole L, Mary Sand Anthony G (2008). The descriptive epidemiology of *Streptococcus pneumoniae* and *Haemophilus influenzae* nasopharyngeal carriage in children and adults in Kilifi. Pediatr Infect Dis J.

[22] Clinical and Laboratory Standards Institute. Performance standards for antimicrobial susceptibility testing. Wayne: Twenty-first Informational Supplement. M100-S24; 2014.

[23] Magiorakos AP (2012). Multidrug-resistant, extensive drug resistant and pandrug-resistant bacteria, an international expert proposal for interim standard definitions for acquired. Clin Microbiol Infect..

[24] Adegbola RA, DeAntonio R, Hill PC, Roca A, Usuf E (2014). Carriage of *Streptococcus pneumoniae* and other respiratory bacterial pathogens in low and lower-middle income countries: a systematic review and meta-analysis. PLoS ONE.

[25] Abdullahi O, Karani A, Tigoi CC, Mugo D, Kungu S (2012). The prevalence and risk factors for pneumococcal colonization of the nasopharynx among children in Kilifi District, Kenya. PLoS ONE.

[26] Menezes P, Azevedo J, Mariela C (2016). Nasopharyngeal carriage of *Streptococcus pneumoniae* among children in an urban setting in Brazil prior to PCV10 introduction. Vaccine.

[27] Soewignjo S, Gessner BD, Sutanto A, Steinhoff M, Prijanto M (2001). Streptococcus pneumoniae nasopharyngeal carriage prevalence, serotype distribution, and resistance patterns among children on Lombok Island, Indonesia. Clin Infect Dis.

[28] Farida H, Severin JA, Gasem MH, Keuter M, Wahyono H (2014). Nasopharyngeal carriage of *Streptococcus pneumonia* in pneumonia-prone age groups in Semarang, Java Island, Indonesia. PLoS ONE.

[29] Vu HT, Yoshida LM, Suzuki M, Nguyen HA, Nguyen CD (2011). Association between nasopharyngeal load of *Streptococcus pneumoniae*, viral coinfection, and radiologically confirmed pneumonia in Vietnamese children. Pediatr Infect Dis J.

[30] Gili R, Meir R, Ron D (2004). Nasopharyngeal carriage of *Streptococcus pneumoniae* by adults and children in community and family settings. Clin Infect Dis..

[31] Rupa V, Isaac R, Manoharan A, Jalagandeeswaran R, Thenmozhi M (2012). Risk factors for upper respiratory infection in the first year of life in a birth cohort. Int J Pediatr Otorhinolaryngol.

[32] O’brien K, Hanna N (2003). Report from a WHO Working Group: standard method for detecting upper respiratory carriage of *Streptococcus pneumonia*. Pediatr Infect Dis J.

[33] Amita J, Pradeep K, Shally A (2005). High nasopharyngeal carriage of drug resistant *Streptococcus pneumoniae* and *Haemophilus influenzae* in North Indian school children. Trop Med Int Health..

[34] Dea M, Cristiana M, Ana-Lu´cia S (2008). Prevalence and risk factors for nasopharyngeal carriage of *Streptococcus pneumoniae* among adolescents. J Med Microbiol.

[35] Eriat M, Chomarat M, Watson M, Garin B (2005). Nasopharyngeal carriage of *Streptococcus pneumoniae* in healthy children, 2 to 24 months of age, in New-Caledonia. Med Mal Infect..

[36] Grant A, Amanda J, Jonathan R, Janelle F, Peter S (2010). Epidemiology of nasopharyngeal carriage of respiratory bacterial pathogens in children and adults: cross-sectional surveys in a population with high rates of pneumococcal disease. BMC Infect Dis.

[37] Ussery X, Gessner B, Lipman H (1996). Risk factors for nasopharyngeal carriage of resistant *Streptococcus pneumoniae* and detection of a multiply resistant clone among children living in the Yukon-Kuskokwim Delta Region of Alaska. Pediatr Infect Dis J..

[38] Chen-Yen K, Kao-Pin H, Yu-Chia H (2011). Nasopharyngeal carriage of *Streptococcus pneumoniae* in Taiwan before and after the introduction of a conjugate vaccine. Vaccine..

[39] Joloba M, Bajaksouzian S, Palavecino E, Whalen C, Jacobs M (2001). High prevalence of carriage of antibiotic-resistant *Streptococcus pneumoniae* in children in Kampala Uganda. Int J Antimicrob Agents.

[40] Donna M, Enoch F, Michael G, Russell W (2002). Nasopharyngeal carriage and susceptibility patterns of *Streptococcus pneumoniae* in Kumasi, Ghana. WAJM..

[41] Susan S, Pak L, Frankie K, Kwok Y, Yu L (2001). Nasopharyngeal carriage of antimicrobial-resistant *Streptococcus pneumoniae* among young children attending 79 kindergartens and day care Centers in Hong Kong. Antimicrob Agents Chemother.

[42] Dagan R, Givon-Lavi N, Zamir O (2002). Reduction of nasopharyngeal carriage of *Streptococcus pneumoniae* after administration of a 9-valent pneumococcal conjugate vaccine to toddlers attending day care centers. J Infect Dis.

[43] Robin E, Alexander M, Loeto M, Keith K (1998). Nasopharyngeal carriage and antimicrobial resistance in isolates of *Streptococcus pneumoniae* and *Haemopbilus influenza* type b in children under 5 years of age in Botswana. Int J Infect Dis..

