# Supplementary material for: Nasopharyngeal carriage of Streptococcus pneumoniae and antimicrobial susceptibility pattern among school children in South Ethiopia: post-vaccination era
Source: BMC Res Notes. 2019 May 29;12:306. doi: 10.1186/s13104-019-4330-0 (PMC6542063; doi:10.1186/s13104-019-4330-0)
Supplement: Supplementary file 1 — Additional file 1: Table S1. Socio-demographic characteristics of school children in Sodo Zuria Woreda, South Ethiopia, 2014. [file 13104_2019_4330_MOESM1_ESM.docx]

**Table S1. Socio-demographic characteristics of school children in** **Sodo Zuria Woreda, South Ethiopia, 2014**

| **Variables** | | **Number (%) of 710)** |
| --- | --- | --- |
| Gender | Male | 356(49.9) (46.2-53.5) |
|  | Female | 358(50.3) (46.5-53.8) |
| Age (95% CI mean )) | 8.06 | 7.88 8.24 |
| Income (parent)  Birr/month | ≤21.7$/month | 264(37.2) |
|  | 21.71$-43.4$  43.41$-65.2$  65.21$-86.9$  >=86.91$ | 202(28.5)  130(18.3)  49(6.9)  65(9.2) |
| Family size(mean(SD)) | 5.08(1.9) |  |
| Numbers of rooms in the house(mean(SD)) | 2.8(1.6) |  |
|  |  |  |
| Number of siblings(mean(SD)) | 2.34(1.5) |  |
|  |  |  |
| Having sibling <6 yrs | Yes | 426(60) |
|  |  |  |
| Co-sleeping with siblings | Yes | 462(65.1) |
|  |  |  |
| Passive smoker | Yes | 105(14.8) |
