# Supplementary material for: Nasopharyngeal carriage of Streptococcus pneumoniae and antimicrobial susceptibility pattern among school children in South Ethiopia: post-vaccination era
Source: BMC Res Notes. 2019 May 29;12:306. doi: 10.1186/s13104-019-4330-0 (PMC6542063; doi:10.1186/s13104-019-4330-0)
Supplement: Supplementary file 2 — Additional file 2: Figure S1. Antibiotic susceptibility pattern of S. pneumoniae strains, Sodo Zuria Woreda, South Ethiopia, 2014. [file 13104_2019_4330_MOESM2_ESM.docx]

**Additional Figure S1. Antibiotic susceptibility pattern of *S. pneumoniae* strains, Sodo Zuria Woreda, South Ethiopia, 2014**
